# Changes in spatiotemporal parameters, joint and CoM kinematics and leg stiffness in novice runners during a high-intensity fatigue protocol

**DOI:** 10.1371/journal.pone.0265550

**Published:** 2022-04-01

**Authors:** Felix Möhler, Cagla Fadillioglu, Thorsten Stein

**Affiliations:** BioMotion Center, Institute of Sports and Sports Science (IfSS), Karlsruhe Institute of Technology, Karlsruhe, Germany; National Tsing Hua University, TAIWAN

## Abstract

Even though running enjoys growing popularity, the effects of fatigue on the running kinematics of novices have rarely been studied. This is surprising, given the risk of running-related injuries when detrimental movement patterns are adopted. Therefore, the goal of the present study was to characterize the effects of fatigue induced by a high-intensity running protocol on spatiotemporal and stiffness parameters as well as on joint kinematics and center of mass (CoM) motion in novice runners. 14 participants performed a standardized treadmill familiarization and ran at 13 km/h until voluntary exhaustion. Kinematics were captured using a 3D motion capture system. Spatiotemporal and stiffness parameters as well as the range of motion (RoM) of the joints and CoM were compared by use of paired t-tests. Time series of the joint angles and CoM motion were analyzed by the statistical parametric mapping method. The results revealed that novice runners did not change spatiotemporal or stiffness parameters, but showed adaptations in joint kinematics, e.g. decreased dorsiflexion and increased pronation in the ankle joint during the swing phase. The findings of this study underline the importance of strengthening the ankle joint to prevent excessive pronation and increase its stability in novice runners.

## 1. Introduction

The World Health Organization recommends a minimum of 150 minutes of aerobic physical activity per week to maintain a healthy life. In this regard, running is a popular activity chosen by several million people in the world from different backgrounds and different expertise levels. Some people, typically referred to as “recreational runners”, utilize running as a complementary activity for maintaining a healthy life, whereas some others, typically referred to as “expert runners” train regularly and systematically and usually to reach an individual performance goal. On the other hand, the term “novice runners” comprises people who are not familiar with running or do not run on a regular basis (e.g. [1]). Beside the popularity of running, the number of running-related injuries is high. Although a direct connection has not yet been clearly defined [2], several studies suggest that injuries are related to atypical foot pronation [2], inadequate hip muscle stabilization [2, 3], overuse [4] or lack of running experience [5]. Novice runners are especially prone to injury, possibly due to one or combination of these factors. A meta-analysis by Videbæk et al. [6] showed that novice runners are at a significantly higher risk of injury than recreational runners. In a study by Kemler et al. [7] analyzing differences in injury risk and characteristics between novice and expert runners, it was reported that incidence of running-related injuries in novice runners was twice as high than in expert runners. It is conceivable that, due to a lack of experience, body awareness among novice runners may be low, leading to an overlooking or misinterpretation of pain signals, particularly under fatigue.

Due to its effectiveness, training under high intensities is common among runners. One popular example is high-intensity interval training (HIIT) [8]. During this type of training, runners perform shorter intervals at a high intensity rather than prolonged jogging at a low intensity. HIIT is widely chosen by not only experienced but also recreational runners to improve performance [9] and shown to be more enjoyable than a moderate intensity continuous exercise [10]. Given the increased injury risk in novice runners, the question arises whether the exhaustion experienced during these training sessions might constitute a risk of injury. A few studies focused on the effects of fatigue induced by a short-distance or a high intensity run. Maas et al. [11] analyzed changes in kinematics in both novice and expert runners (mean time to exhaustion 1693 ± 588 s (~ 28.2 ± 9.8 min) and 947 ± 284 s (~ 15.8 ± 4.7 min), respectively) and reported that novices had larger kinematic adjustments compared to experts. These kinematic adjustments involved hip abduction, ankle plantar flexion, and kinematics of the pelvis and trunk. By analyzing kinematic changes after running-induced fatigue (mean time to fatigue 19.7 ± 7.8 min), Koblbauer et al. [12] found changes in trunk motion and ankle eversion in novice runners. Yu et al. [13] showed significant effects of running induced fatigue on the lower limbs mechanics. However, none of these studies analyzed the effects of a high-intensity run with a shorter duration (<10 min). Furthermore, intensity was shown to effect the reaction to fatigue as shown by different reactions evoked by different fatigue protocols [14]. Maas et al. [11] showed that novice runners react differently to fatigue than more experienced runners during low to moderate intensity runs. Novice runners showed an increase in trunk forward lean which was not seen in expert runners and an increase in hip abduction during mid-swing, whereas a decrease was found for expert runners. This indicates that fatigue effects found in expert runners [15, 16] are not directly transferable to novice runners. In experienced runners, effects of fatigue on spatiotemporal and stiffness parameters [15, 17], on joint kinematics and kinetics [18] and on leg symmetry [19] were found. However, the fatigue effects induced by a high-intensity run in novice runners have not gained much attention yet. Therefore, research is required to understand how high-intensity fatigue affects spatiotemporal and stiffness parameters and joint kinematics in novice runners. The spatiotemporal parameters are the fundamental parameters for locomotion analyses, which may help to understand the running style [20], whereas the stiffness parameters may reveal clues on the characteristics of the body regarding storage and return of elastic energy during the support phase [21]. Finally, the analysis of joint kinematics and center of mass (CoM) motion in 3D provides a deeper understanding of adaptations of the body in different joints and planes. Besides the mean values, consideration of the coefficient of variation (CV) helps to understand the influences of fatigue on movement variability [15, 22, 23].

In summary, despite its practical relevance, the effects of fatigue on kinematics induced by high-intensity running have not yet been considered for novice runners. The goal of the present study was to analyze the effects of fatigue induced by a high-intensity run on spatiotemporal parameters, leg and vertical stiffness, 3D joint kinematics as well as the CoM trajectory in novice runners. In addition, this study aimed to conduct an explorative analysis of entire joint angle and CoM time series data by means of statistical parametric mapping (SPM). Since novices were found to show stronger reactions to fatigue compared to experts after a run to fatigue at a moderate intensity [11] and as we found marked effects of fatigue in experienced runners in our previous study [24], we hypothesized that novices would show pronounced reactions to fatigue in both joint kinematics and spatiotemporal and stiffness parameters. The results from this study may be helpful to understand typical adaptation strategies of novice runners to the fatigue induced by running at a high intensity.

## 2. Materials and methods

### 2.1 Participants

Fourteen healthy male novice runners participated in the study (Table 1). Inclusion criteria were: participating in a sports activity once or twice a week, a BMI between 19 and 23 kg/m^2^ and no regular running activity, specifically no more than one run per month. Exclusion criteria were: recent injuries or pain and performing regular or systematic running training. All participants provided written informed consent and the study was approved by the ethics committee of the Karlsruhe Institute of Technology.

**Table 1:** Sample characteristics (mean ± standard deviation).

Participants [n]	14
Age [years]	27 ± 4
Body weight [kg]	77.5 ± 10.3
Body height [m]	1.82 ± 0.06
Running activity [min/week]	14 ± 18
Other sports activity [min/week]	110± 71

### 2.2 Experimental design

**Fig 1:**
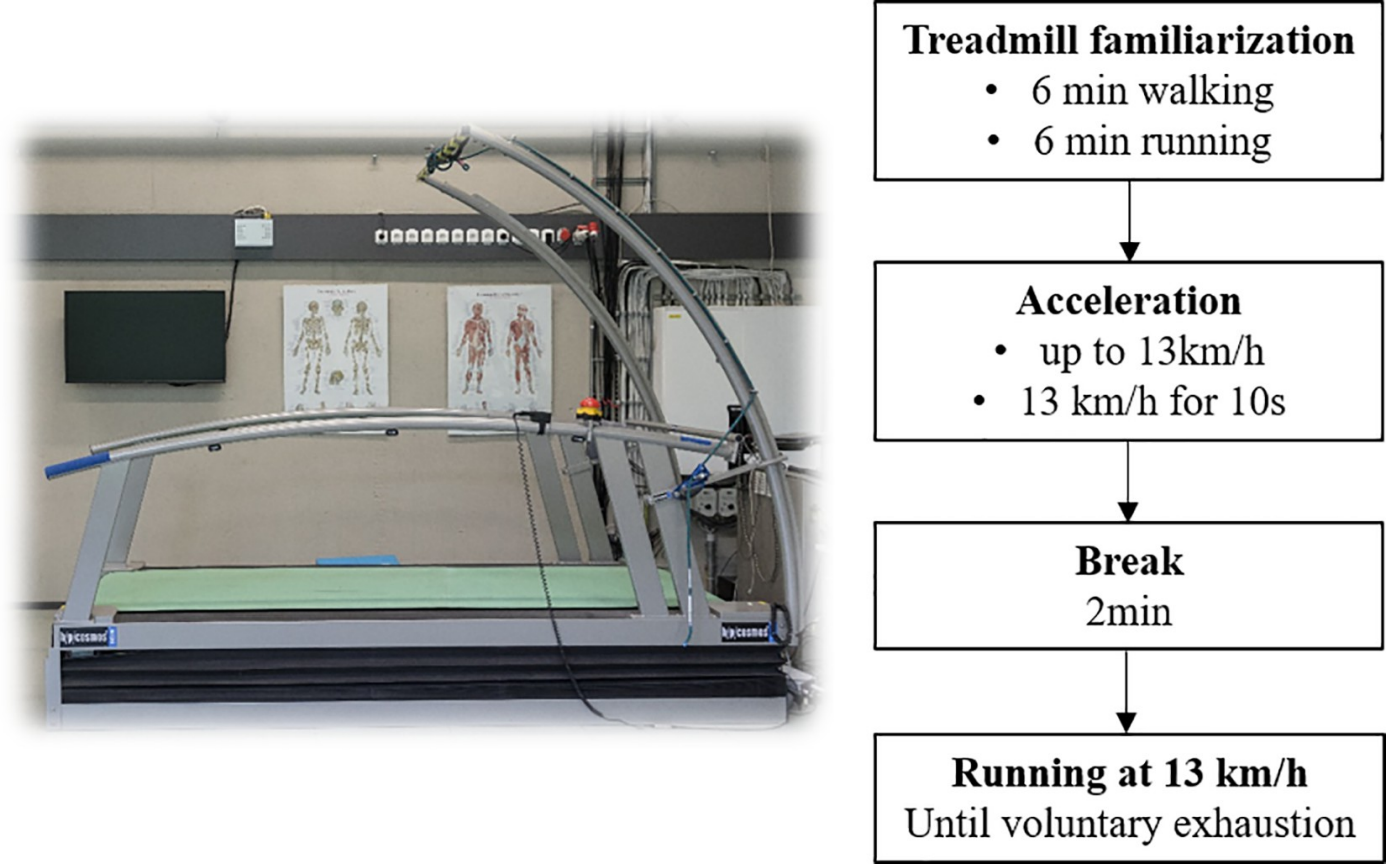
Illustration of the experimental design and picture of the treadmill used in the study.

The experimental design is illustrated in Fig 1. The experiment was conducted on a motorized treadmill (h/p/cosmos Saturn, Nussdorf-Traunstein, Germany) with a slope of 1% [25]. After a standardized treadmill familiarization (6 min walking, 6 min running [26, 27]), the treadmill was accelerated up to the test speed of 13 km/h. This speed was held for 10 seconds. Then, participants had a break of 2 min before the actual measurement. During the measurement, participants ran at a fixed speed of 13 km/h until voluntary exhaustion with their own shoes. This speed was chosen based on previous tests performed with novice runners that did not participate in this study. This speed was found to be appropriate for an exhaustion that sets in after 5–7 minutes for male novice runners. After voluntary exhaustion was reached, participants were asked to rate their perceived exertion using the Borg scale [28]. Participants were instructed to look ahead during the run. For safety reasons, participants were held by a safety harness which was connected to an emergency-off.

### 2.3 Data acquisition and processing

Kinematic data were recorded at 200 Hz using 16 Vicon cameras (Vicon Motion Systems, Oxford Metrics Group, Oxford, UK) throughout the whole run. A total of 42 reflective markers were attached to the participants’ skin and anthropometric measures were taken according to the instructions of the Alaska Dynamicus modelling system (Advanced Lagrangian Solver in Kinetic Analysis, Insys GmbH, Chemnitz, Germany; [29]).

After preprocessing the marker data using Vicon Nexus 2.11.0 software, all further data processing was performed in Matlab R2020b (The MathWorks, Natick, MA, USA). Marker data were filtered using a second order 15 Hz low-pass Butterworth filter. An inverse kinematics calculation was performed using the ALASKA Dynamicus modelling system [29].

Gait events were identified using the change of sign of the heel or forefoot markers, and the vertical acceleration of the toe marker for heel strike and toe-off, respectively [30]. 20 consecutive gait cycles at the beginning of the run (PRE) and 20 gait cycles at the end of the run (POST) were further analyzed. These gait cycles were time normalized to 101 time points each.

Similar to our previous work in expert runners [24], we analyzed time of stance (right foot strike to right toe-off), time of flight (right toe-off to left foot strike) and the stride frequency (right foot strikes per second), since these spatiotemporal parameters are used to generally characterize running gait. Vertical and leg stiffness were shown to be influenced by fatigue [17, 31] and were therefore also included in our analysis. Additionally, alterations in spatiotemporal parameters might be explained by concurrent alterations in stiffness parameters. Due to a lack of ground reaction force data, stiffness was calculated following the method evaluated by Morin et al. [32]. Besides mean values and standard deviations, we also calculated CVs to quantify changes in variability of the running movement.

The aim of our study was to reveal the effects of fatigue on running kinematics in novices in an explorative manner. We therefore performed a time series analysis by means of SPM [33] in all three planes (sagittal (S), frontal (F), and transverse (T)) on the relevant joints in the lower limb (ankle, knee, and hip) and in the torso (lumbar and thoracic spine). Additionally, range of motion (RoM) was calculated separately for the stance and the flight phases. A higher RoM, as a measure of limits of motion, could be used as an indicator for a higher risk of soft tissue injury due to the higher stresses. The trajectory of the center of mass (CoM) was analyzed in the same way as the joint angles by performing a time series analysis and calculating the RoM since the transportation of the CoM is the main goal of locomotion [20]. The data can be found in the supplemental material.

### 2.4 Statistics

To analyze the spatiotemporal parameters, their CVs and RoM, the results for the 20 gait cycles per condition were averaged for each participant for subsequent statistical analysis. Normality of distribution was tested using the Shapiro-Wilk test. When distribution was normal, these parameters were compared using paired t-tests. When distribution was non-normal, a Wilcoxon signed rank test was used. Due to the non-normal distribution of part of the data, the robust d (d_r_) was calculated. This is done following the regular calculation of Cohen’s d while taking the 20% trimmed mean and in the 20% winsorized variance. Thereby, 0.2 < d_r_ ≤ 0.5 was interpreted as a small effect, 0.5 < d_r_ ≤ 0.8 as a medium effect and d_r_ > 0.8 as a large effect [34].

The time-normalized joint angle and CoM time series were compared using statistical non-parametric mapping (www.spm1d.org) due to non-normal distribution of the data. It was assumed that both legs would fatigue at a similar rate [35], thus analyses were performed for the right side only. For all statistical analyses, the level of significance was set *a priori* to p = 0.05.

## 3. Results

### 3.1 Spatiotemporal parameters, vertical and leg stiffness and their variability

The participants stopped running after 6.18 ± 2.45 minutes, and exhaustion was confirmed by a Borg scale rating of 18.7 ± 1.0 which corresponds to “very very hard” [28]. Effects of fatigue were not apparent in stance time, flight time, stride frequency, vertical stiffness or leg stiffness, nor in their CVs (Table 2). However, joint angle RoMs and time courses showed pronounced effects of fatigue.

**Table 2:** Mean ± standard deviation of spatiotemporal parameters, vertical and leg stiffness along with their corresponding coefficients of variation (CVs).

	PRE	POST	p	d_r_
Time of support [s]	0.25 ± 0.02	0.25 ± 0.03	0.617	0.124
Time of flight [s]	0.20 ± 0.05	0.21 ± 0.04	0.301	0.189
Stride frequency [1/s]	1.43 ± 0.05	1.41 ± 0.06	0.087	0.557
Vertical stiffness [kN/m]	8.82 ± 1.83	9.02 ± 2.20	0.485	0.177
Leg stiffness [kN/m]	6.16 ± 1.42	6.31 ± 1.69	0.452	0.176
*Coefficients of variation*				
Time of support	0.02 ± 0.01	0.03 ± 0.01	0.761^nnd^	0.115
Time of flight	0.06 ± 0.03	0.06 ± 0.02	0.855 ^nnd^	0.248
Stride frequency	0.01 ± 0.01	0.02 ± 0.01	0.450	0.314
Vertical stiffness	0.06 ± 0.02	0.06 ± 0.02	0.715 ^nnd^	0.166
Leg stiffness	0.06 ± 0.02	0.07 ± 0.02	0.670 ^nnd^	0.149

p-values as calculated by the paired t-tests and effect sizes are given. d_r_ values of 0.2–0.50, 0.5–0.8 and > 0.8 indicate small, medium and large effects, respectively. A superscript “nnd” behind the p-value signifies a non-normal distribution.

### 3.2 Analyses of range of motion of joint and CoM movements

The joint angle RoMs showed pronounced effects of fatigue, especially during stance phase. In the upper body, changes occurred in all three planes, whereas the sagittal and frontal planes were most affected in the lower limbs. All changes in RoM with fatigue were increases (Table 3). Results for the stance and flight phase are presented in separate paragraphs. The difference between the PRE and POST mean value (ΔPOST_PRE) is given in brackets. A positive value signifies an increase from PRE to POST.

**Table 3:** Mean ± standard deviation of the RoM of joints in degrees (°) and of the CoM in mm are shown for stance and flight phases separately.

	PRE	POST	p	d_r_
*Stance phase*				
Ankle–S [°]	48.62 ± 4.18	49.28 ± 4.33	0.199	0.284
Ankle–F [°]	15.86 ± 3.94	15.35 ± 4.12	0.259	0.157
Ankle–T [°]	10.56 ± 2.84	10.70 ± 3.03	0.419	0.064
**Knee–S [°]**	**33.57 ± 4.52**	**37.68 ± 4.50**	**< 0.001**	**1.605**
Knee–F [°]	5.00 ± 2.42	5.83 ± 2.04	0.068*	0.588
Knee–T [°]	10.89 ± 3.88	9.49 ± 3.36	0.244	0.544
**Hip–S [°]**	**46.70 ± 5.13**	**49.59 ± 5.45**	**< 0.001**	**0.842**
**Hip–F [°]**	**16.65 ± 3.44**	**18.86 ± 4.39**	**0.007**	**0.602**
Hip–T [°]	11.36 ± 3.77	12.61 ± 2.97	0.071	0.319
**Lumbar spine–S [°]**	**9.89 ± 2.21**	**10.97 ± 2.94**	**0.020** ^**nnd**^	**0.802**
**Lumbar spine–F [°]**	**8.27 ± 1.77**	**10.47 ± 1.83**	**< 0.001**	**1.785**
**Lumbar spine–T [°]**	**5.61 ± 0.95**	**6.99 ± 1.41**	**< 0.001**	**2.059**
**Thoracic spine–S [°]**	**5.00 ± 1.06**	**5.87 ± 1.58**	**< 0.001** ^**nnd**^	**1.363**
**Thoracic spine–F [°]**	**15.46 ± 2.14**	**20.01 ± 3.01**	**< 0.001**	**3.773**
**Thoracic spine–T [°]**	**32.71 ± 6.66**	**39.97 ± 9.10**	**< 0.001**	**1.660**
COM ant-post [mm]	13.58 ± 1.61	15.30 ± 3.16	0.054	0.762
**COM med-lat [mm]**	**6.46 ± 2.08**	**8.62 ± 2.32**	**0.009**	**1.328**
COM vertical [mm]	68.96 ± 7.38	71.17 ± 8.79	0.243	0.319
*Flight phase*				
Ankle–S [°]	6.71 ± 2.88	5.76 ± 2.86	0.173 ^nnd^	0.483
Ankle–F [°]	3.18 ± 1.27	2.73 ± 1.64	0.258	0.359
**Ankle–T [°]**	**2.14 ± 1.29**	**2.73 ± 1.64**	**0.009** ^**nnd**^	**0.696**
Knee–S [°]	44.66 ± 9.68	45.61 ± 10.34	0.389	0.151
Knee–F [°]	5.08 ± 2.19	5.69 ± 3.15	0.2142	0.225
**Knee–T [°]**	**6.91 ± 2.81**	**8.87 ± 3.51**	**0.002** ^**nnd**^	**0.904**
Hip–S [°]	6.95 ± 2.94	6.21 ± 2.67	0.179	0.282
**Hip–F [°]**	**6.59 ± 2.49**	**7.54 ± 2.58**	**0.009**	**0.489**
Hip–T [°]	8.46 ± 3.40	9.23 ±3.74	0.296*	0.216
Lumbar spine–S [°]	4.88 ± 1.51	5.34 ± 2.07	0.208	0.316
**Lumbar spine–F [°]**	**2.64± 1.20**	**3.34 ± 1.19**	**0.002**	**0.640**
Lumbar spine–T [°]	1.48 ± 0.40	1.56 ± 0.47	0.204	0.206
**Thoracic spine–S [°]**	**2.59 ± 0.71**	**2.92 ± 1.01**	**0.048**	**0.427**
Thoracic spine–F [°]	4.19 ± 1.26	4.54 ± 1.32	0.129	0.334
Thoracic spine–T [°]	8.43 ± 2.07	8.86 ± 2.69	0.147	0.233
COM ant-post [mm]	8.30 ± 2.49	8.75 ± 2.29	0.235	0.343
**COM med-lat [mm]**	**3.47 ± 1.09**	**4.72 ± 1.18**	**< 0.001**	**1.323**
COM vertical [mm]	25.32 ± 7.36	27.81 ± 7.75	0.051	0.331

p-values as calculated by the paired t-test and Cohen’s d as effect sizes are also given. Bold font indicates significant differences (p < 0.05). d_r_ values of 0.2–0.50, 0.5–0.8 and > 0.8 indicate small, medium and large effects, respectively. A superscript “nnd” behind the p-value signifies a non-normal distribution. S, F and T signify the sagittal, frontal and transverse planes, respectively.

#### Stance phase

The RoM increased significantly in the knee joint in the sagittal plane with a high effect size (knee: ΔPOST_PRE_S_ = 4.11°), in the hip joint in the sagittal plane with a high effect size, and in the frontal plane with a medium effect size (hip: ΔPOST_PRE_S_ = 2.89°; ΔPOST_PRE_F_ = 2.21°). In the lumbar and thoracic spine, the RoM significantly increased in all three planes with high effect sizes (lumbar spine: ΔPOST_PRE_S_ = 1.08°; ΔPOST_PRE_F_ = 2.2°; ΔPOST_PRE_T_ = 1.38°; thoracic spine: ΔPOST_PRE_S_ = 0.87°; ΔPOST_PRE_F_ = 4.55°; ΔPOST_PRE_T_ = 7.26°). The RoM of the CoM significantly increased in the medio-lateral direction with a high effect size (ΔPOST_PRE_medio-lateral_ = 2.16 mm).

#### Flight phase

There were fewer changes in the flight phase compared to stance. RoM increased significantly in the ankle and knee joints in the transverse plane with a medium and with a high effect size, respectively (ankle: ΔPOST_PRE_T_ = 0.59°, knee: ΔPOST_PRE_T_ = 1.96°). In the hip joint and in the lumbar spine RoM increased significantly in the frontal plane with medium effect sizes (hip: ΔPOST_PRE_F_ = 0.95°; lumbar spine: ΔPOST_PRE_F_ = 0.7°). The RoM of the thoracic spine significantly increased in the sagittal plane with a medium effect size (ΔPOST_PRE_S_ = 0.33°). The RoM of the CoM significantly increased in the medio-lateral direction with a high effect size (ΔPOST_PRE_medio-lateral_ = 1.25mm).

### 3.3 Time series analyses of joint and CoM movements

**Fig 2:**
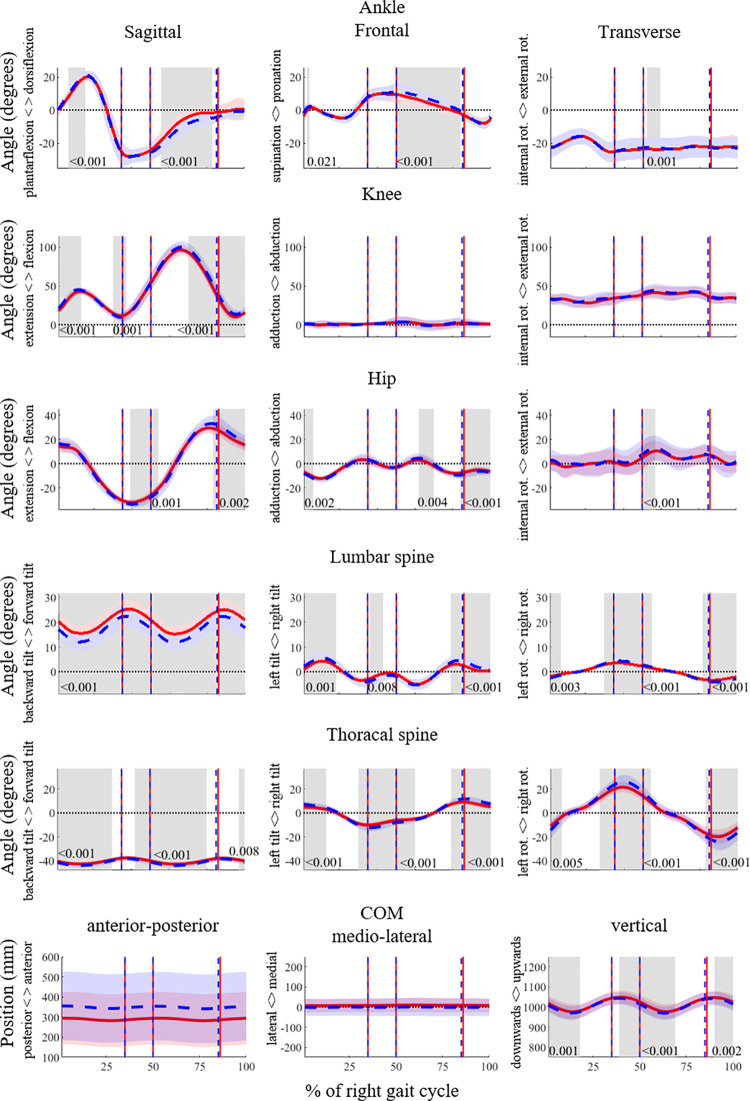
Time series of the joint angles and the CoM. SPM analyses for the angles of the ankle, knee, hip (right side), lumbar spine and thoracic spine in degrees and of the trajectory of the center of mass (CoM) in mm for the entire running gait cycle (from right foot strike to right foot strike) in 3D. The PRE and POST time series data are shown in red and blue, respectively. Significant differences (p < 0.05) are highlighted with grey areas and corresponding p-values are given. RTO signifies right toe-off, LFS left foot strike and LTO left toe-off.

The time series of the joint angles and the CoM, and the results of the SPM analysis are shown in Fig 2. The participants showed a higher dorsiflexion during stance in the POST and more plantarflexion during swing. Around heel strike, the knee joint showed greater flexion in the POST. The hip joint was more extended in the POST after toe-off and more flexed before right heel strike. In the POST, the thigh was more abducted during swing and more adducted around heel strike.

In the POST, participants showed a greater backward lean in the lumbar spine. Around the right heel strike, participants were more tilted to the right and rotated to the left. In the flight phase after right toe-off, participants rotated more to the right. For the left heel strike and left toe-off, the reactions were an increased tilt to the left and an increased rotation to the left. In the thoracic spine, there was a greater backward lean in the POST compared to PRE. Analogously to the effects seen in the lumbar spine, participants tilted to the right and rotated to the left during right heel strike and left toe-off. During right toe-off and left heel strike, runners tilted to the left and rotated to the right.

The CoM showed a significantly lower trajectory in the POST, mainly after right and left heel strike. In the other directions, there were no significant differences between the PRE and the POST trajectories of the CoM.

The joint angle time series of the lower limbs showed significant effects of fatigue in the frontal and sagittal planes. The lumbar and thoracic spine were affected in all three planes. The CoM motion was affected in the vertical direction, showing a lower trajectory.

## 4. Discussion

The aim of this study was to characterize the effects of fatigue induced by a high-intensity run on the running kinematics of novices. We hypothesized that we would find pronounced reactions to the induced fatigue across classical biomechanical parameters used for the analysis of running. Since we found effects of fatigue on joint kinematics, but not on the spatiotemporal parameters or stiffness and their CVs, our hypothesis could only be partly accepted.

### 4.1 Spatiotemporal parameters, vertical and leg stiffness and their variability

Similar to expert runners, novice runners kept their stride frequency constant (~85.2 strides/min). This value is very close to the mean metabolically optimal stride frequency (84.8 ± 3.6 strides/min) reported by Lieberman et al. [36], who analyzed the effects of stride frequency and foot position at landing on a variety of biomechanical parameters. Nevertheless, the lack of change in the other spatiotemporal and stiffness parameters and CVs was inconsistent with changes found in expert runners [15]. The increase in time of support and the decrease in time of flight were interpreted as a shock attenuation strategy [14]. This strategy is not observable in our sample. This finding could be explained by the fact that novice runners lack strategies to maintain the given running speed and thus ended the run earlier than expert runners, despite the high Borg scale rating. Analyzing the coordination between joint angles could provide deeper insights [37, 38] and therefore reveal further insights into possible compensation mechanisms.

### 4.2 Analyses of range of motion of joint and CoM movements

As in our previous study [24], all significant changes in RoM were increases from PRE to POST and occurred mainly in the stance phase. Changes in the lower extremities happened mainly in the sagittal and frontal planes at the knee and hip. The observed increases in RoM could be caused by attempts to generate the torque necessary to keep up with the treadmill speed. The increase in RoM in the hip joint, evoked through a greater abduction around heel strike followed by an increased adduction at the end of stance phase, might be caused by an increased need of shock absorption [39]. These increases in RoM at the hip and knee could present increased injury risk, since the tissues are more stretched. Maas et al. [11] also showed an increased RoM of the pelvis rotation for both novice and expert runners after an exhaustive run with a longer duration (mean time to exhaustion 1,693 ± 588 s (~ 28.2 ± 9.8 min) and 947 ± 284 s (~ 15.8 ± 4.7 min), respectively). Further studies should try to reveal where these increases might reach a clinical relevance. Increases in upper body RoM (lumbar and thoracic spine) were even more pronounced than in experienced runners, despite the lower running speed [24], and are a sign of compensatory movements of the trunk [40] which could be due to an insufficient core musculature. The RoM of the CoM trajectory in novice runners was greater in the horizontal plane in the POST state, but the RoM of their vertical displacement was not affected. This is in line with the lack of change in stiffness parameters.

### 4.3 Time series analyses of joint and CoM movements

To broaden our analysis from only discrete parameters, we performed a time series analysis by means of SPM. Several joint angles showed significant effects of fatigue. Even though the novice runners may lack strategies to keep up a certain running speed when fatigued, they may instinctively try to reduce their energy expenditure by adapting their running style accordingly.

The ankle joint showed an increased plantarflexion and pronation throughout the swing phase, may be an indicator of an unstable ankle joint due to fatigued lower leg muscles, especially the tibialis anterior [23, 41, 42]. The lower plantarflexion around toe-off can be interpreted as an effort to save energy since running economy was reported to be strongly related to a less extended leg at toe-off, which can be achieved through greater knee-flexion [43]. Similarly, a greater flexion of the hip and knee also indicate that the leg was less extended in the POST, potentially trying to maximize force production [43] to keep the speed constant under fatigue. According to Moore et al. [44], less leg extension would allow the leg extensor muscles to produce a higher level of propulsive force by operating at a more favorable position on the force-length curve. The increases in flexion in the legs and trunk could be interpreted as a shock attenuation strategy [14]. Furthermore, a less extended leg may reduce the amount of energy needed for flexing the leg in the swing phase, which ultimately would result in a decreased level of energy cost [43].

In addition to the changes in the lower limbs, the upper body showed pronounced reactions to fatigue. The changes in the lumbar and thoracic spine showed that runners rounded their spine more. These changes in upper body posture together with the increased flexion of the leg could explain the lower CoM in the POST. The strong increases in upper body inclination and rotation (in both the lumbar and the thoracic spine) indicate that the runners are not able to stabilize their trunk to counteract the torques induced by the running motion [40].

### 4.4 Limitations

A fixed fatigue speed was chosen for all participants. Even though this speed was possibly not equally demanding for all participants, all participants were considerably fatigued as shown by the Borg scale rating of “very very hard”. Therefore, it is supported that all participants underwent an exhausting high-intensity run. However, it would have been preferable to choose an individualized fatigue speed, e.g. based on a previous lactate threshold testing instead of choosing a fixed speed based on previous tests. Even though running on a treadmill and running overground show differences [45], treadmill running was preferred in this study to avoid mixing the effects of fatigue and running speed [46]. It would have been preferable to have chosen the sample size based on an a priori power calculation although the sample size corresponds to common sample sizes [14]. Additionally, only male participants were chosen to keep the sample homogenous. Accordingly, it should be noted that the results are not necessarily transferable to female runners [47].

### 4.5 Conclusion

To the best of our knowledge, this was the first study investigating the effects of fatigue induced by a high-intensity running protocol in novice runners. The results revealed that novice runners showed pronounced adaptations in joint kinematics. However, there were no changes in their spatiotemporal and stiffness parameters, possibly because novice runners do not have adequate strategies to keep up a fixed running speed in an exhausted state. The changes observed in the joint kinematics, especially in foot pronation and hip stabilization, showed patterns associated with running-related injuries [2, 42]. From an injury prevention point of view, training programs designed for running novices should therefore involve strengthening the ankle joint to increase the ankle joint stability [23], as well as of core musculature to establish a more stable trunk during running. From a performance point of view, novice runners should train at high intensities to develop strategies to maintain a high speed even in a fatigued state.

## Supporting information

S1 Data (MAT)
